# Anti-OLA1 autoantibody is a potential early diagnostic marker for hepatocellular carcinoma

**DOI:** 10.3389/fimmu.2025.1647809

**Published:** 2025-10-30

**Authors:** Wenzhuo Xiong, Xuehui Duan, Liping Dai, Peng Wang, Hua Ye, Jianxiang Shi, Keyan Wang

**Affiliations:** ^1^ Henan Institute of Medical and Pharmaceutical Sciences, Zhengzhou University, Zhengzhou, China; ^2^ Henan Key Laboratory of Tumor Epidemiology & Henan International Joint Laboratory of Tumor Biomarkers and Molecular Imaging, Zhengzhou University, Zhengzhou, China; ^3^ National Key Laboratory of Metabolism Disorder and Esophageal Cancer Prevention & Treatment, Zhengzhou University, Zhengzhou, China; ^4^ Department of Epidemiology and Health Statistics, College of Public Health, Zhengzhou University, Zhengzhou, China

**Keywords:** HCC, autoantibody, diagnostic, OLA1, plasma

## Abstract

**Background:**

Early detection of hepatocellular carcinoma (HCC) enhances survival outcomes. Tumor-associated autoantibodies demonstrate early emergence during carcinogenesis, offering potential as non-invasive diagnostic biomarkers. This multicenter study aims to evaluate the diagnostic value of anti-OLA1 autoantibody in HCC.

**Methods:**

Protein microarray was used to screen for autoantibodies in AFP - negative patients with HCC (ANHCC) and normal controls (NC) during the discovery stage. In the validation stage, 413 HCC patients and 655 control from three centers were recruited to evaluate anti-OLA1 autoantibody performance using enzyme-linked immunosorbent assay. Receiver Operating Characteristic (ROC) curve analysis and area under the curve (AUC) were used to assess its diagnostic value. Anti-OLA1 autoantibody was combined with liver function parameters in a logistic regression model to improve HCC diagnosis. Finally, OLA1 expression, immune infiltration, and prognostic impact were analyzed using public databases.

**Results:**

Anti-OLA1 autoantibody was identified by protein microarray with an AUC of 0.75 for distinguishing ANHCC from NC. Multi-center validation confirmed these results, showing AUCs from 0.607 to 0.713 and sensitivity from 18.8% to 35.2%. Incorporating liver function parameters significantly improved diagnostic efficiency, with a net reclassification index of 1.04 and an integrated discrimination index of 0.46 in Zhengzhou, validated by an AUC of 0.93 in Nanchang. Public database analysis revealed OLA1 overexpression in HCC tissues correlates with increased immune cell infiltration and predicts poor early prognosis (*P* < 0.05).

**Conclusion:**

Anti-OLA1 autoantibody shows promise as a serological HCC biomarker, with diagnostic performance significantly enhanced through combination with routine liver function parameters.

## 1 Introduction

Hepatocellular carcinoma (HCC) represents a significant global health challenge and plays a crucial role within the spectrum of cancer-related diseases. In 2022 a total of 870, 000 new HCC cases were diagnosed worldwide and 760, 000 deaths, ranking third among cancer-related mortalities (1). This situation is particularly severe in China, where the 5-year survival rate for HCC patients was only 12.1% in 2020 (2). Early diagnosis coupled with prompt treatment can significantly enhance the prognosis of HCC and the quality of patient’s life, while alleviating the burden on patients and their families.

Early diagnostic methods, such as serum Alpha-Fetoprotein (AFP), liver ultrasound imaging, and histological examination, have improved the diagnostic efficiency for HCC (3–5). However, given that approximately 40% of HCC patients exhibit AFP negativity (≤20 ng/mL), abdominal ultrasound demonstrates limited sensitivity in the early detection of HCC, and liver tissue biopsy is associated with high cost and risk in clinical practice, the early diagnosis of HCC continues to encounter substantial challenges (6–8).

Autoantibodies, serving as tumor markers, offer several advantages for early diagnosis in HCC: they can be detected years prior to clinical manifestation, exhibit remarkable stability in serum for up to 3 months, and demonstrate higher titers than their target antigens, enabling easy detection by Enzyme-linked immunosorbent assay (ELISA) (9–11). This noninvasive detection method optimizes patient comfort while promoting the early detection of cancer to a significant extent.

OLA1 (Obg Like ATPase 1) protein is a highly conserved ATP/GTPase. At the molecular level, downregulation of OLA1 leads to G0/G1 phase arrest and triggers significant apoptosis, indicating that OLA1 interacts with P21 and enhances CDK2 expression, thereby promoting the progression of HCC (12). However, there is currently no research investigating the potential of anti-OLA1 autoantibody as an early diagnostic marker for HCC.

Initially, via protein microarray detection in the present study, it was found that the level of anti-OLA1 autoantibody in the serum of patients with ANHCC—was significantly higher than that in healthy controls. Subsequently, the validation and evaluation of anti-OLA1 autoantibody as an early diagnostic marker for HCC was conducted in a multiple clinical center by ELISA. In addition, by searching and analyzing public databases, the possible impact of high expression of OLA1 on HCC was explored.

## 2 Patients and methods

### 2.1 Serum samples

The serum samples utilized in this study were obtained from the serum bank of the Tumor Epidemiology Laboratory at Zhengzhou University. HCC patients were diagnosed following the 2017 criteria established in China and staged adhering to the 8th edition of the AJCC Cancer Staging Manual. Normal Controls (NC) exhibited no evidence of liver diseases, autoimmune disorders, excessive alcohol use, or personal history of cancer. Serum samples were collected from patients prior to the initiation of any treatment. All subjects signed informed consent forms, and the study received approval from the Ethics Review Committee of Zhengzhou University (ZZURIB2019-001).

### 2.2 Protein microarray

The protein chip used in this study contains 154 proteins or peptide segments, of which 143 are encoded by the cancer driver gene reported by Vogelstein et al. (13). This chip was specifically designed to screen tumor-associated antigens (TAAs). The detailed protocol can be found in a previous study (14). The intensity of the signal represents the abundance of autoantibodies that bind to specific proteins. In order to minimize the bias caused by background signals, the signal-to-noise ratio (SNR) was used in the following analysis as the ratio of foreground-to-background intensity for each protein. To clarify the diagnostic value of autoantibodies on the chip for ANHCC, first, ROC curves of each candidate autoantibody on the chip were analyzed to distinguish between ANHCC patients and NC. Subsequently, all candidate autoantibodies were sorted in descending order of their AUC values. Results showed that the anti-OLA1 autoantibody exhibited the highest AUC value among all candidate indicators, indicating that this antibody could serve as the promising biomarker for ANHCC diagnosis.

### 2.3 Enzyme-linked immunosorbent assay

The OLA1 recombinant protein was purchased from Yunclone Company (Wuhan, China). The concentration, purity, and molecular weight of the OLA1 protein were confirmed by SDS-PAGE gel electrophoresis. A 96-well microplate was coated overnight at 4°C with recombinant OLA1 protein at a concentration of 0.5μg/mL. The serum samples were diluted with 1% BSA at a ratio of 1:100, and the secondary antibody was diluted at a ratio of 1:10, 000. A detailed protocol can be found in our previous articles (15). The optical density (OD) values were measured at wavelengths of 450nm and 620 nm. Each microtiter plate included two blank controls and eight identical mixed sera samples, which serve as quality controls for monitoring the consistency and reliability of ELISA assays.

### 2.4 Statistical analysis

Statistical analyses were conducted using GraphPad Prism 8.0, IBM SPSS 21.0, and R 4.3.3 software. Receiver operating characteristic (ROC) curve analysis was performed to evaluate diagnostic value, with sensitivity, specificity, positive predictive value (PPV), negative predictive value (NPV), and accuracy calculated to assess the validity and reliability of OLA1 autoantibody across different clinical characteristics. For comparisons of signal-to-noise ratio (SNR) or optical density (OD) values, the Mann-Whitney U test was used for two-group analyses, while the Kruskal-Wallis H test was applied for multiple-group comparisons. The Pearson chi-squared test was utilized to compare autoantibody frequencies between different groups within each dataset. A *P*-value< 0.05 was considered statistically significant.

For the combined evaluation of autoantibodies and liver function parameters via logistic regression modeling: Liver function parameters were first preprocessed with log_2_ transformation to satisfy model assumptions. A stepwise foward method was then employed to sequentially incorporate variables into the model, followed by the construction of a combined model. The aforementioned Logistic regression analysis, along with collinearity assessment and event per variable (EPV) ratio calculation, were all performed using IBM SPSS Statistics 21.0.

## 3 Result

### 3.1 Serum samples and study design

**Figure 1:**
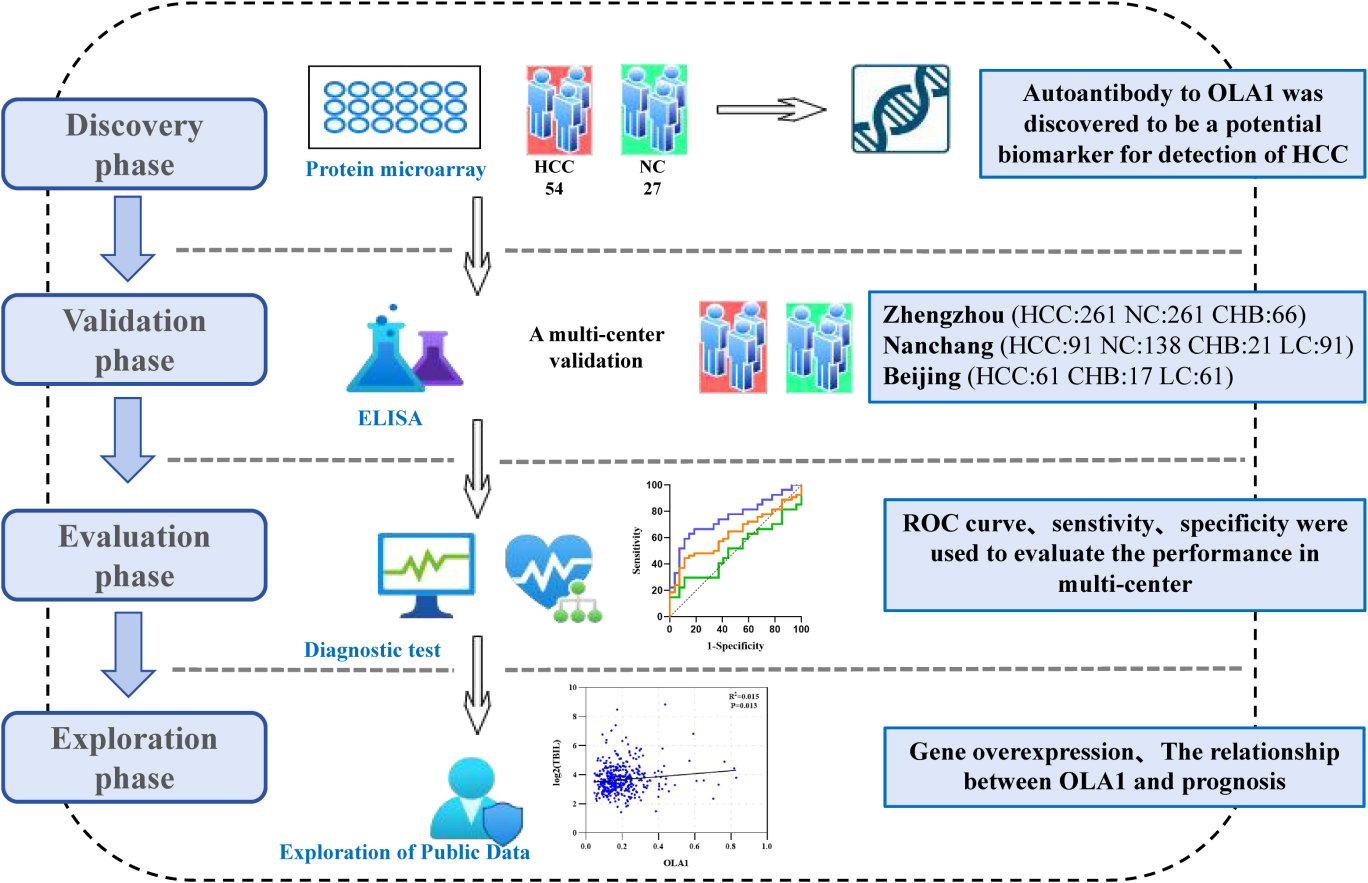
The flow diagram of this study. This study was mainly divided into discovery phase, validation phase, evaluation phase and Exploration phase. HCC, hepatocellular carcinoma; NC, normal control; CHB, Chronic hepatitis B; LC, Liver Cirrhosis.

Our study is divided into four distinct phases: discovery phase, validation phase, evaluation phase, and exploration phase (**Figure 1**). In the discovery phase, 81 serum samples were selected for biomarker screening using protein microarray. Among these samples, 54 were obtained from patients suffering from HCC, while 27 were from healthy control subjects. We focused on the analysis of autoantibody expression profiles and relevant statistical data in ANHCC patients. With healthy individuals as controls and the AUC value for ANHCC diagnosis as the evaluation metric, the anti-OLA1 autoantibody with the highest AUC value was ultimately identified as the core indicator for HCC diagnosis (**Supplementary Table S1**). During the validation phase, we expanded our sample size to include 1068 samples from three regions: 588 samples from Zhengzhou center, 341 samples from Nanchang center, and 139 samples from Beijing center. Detailed demographic and clinical characteristics of these samples, including age, gender, disease stage, and other relevant factors, are summarized in **Table 1**. During the evaluation stage, a clinical subgroups analysis was conducted on serum samples from the validation phase. This analysis aimed to assess the diagnostic performance of autoantibodies across genders, ages, and disease stages, as well as its correlation with liver function parameters. Finally, In the exploration stage, we investigated the association between elevated anti-OLA1 autoantibody and OLA1 expression from various angles.

**Table 1:** Baseline characteristics of participants.

Clinical characteristics	Discovery phase		Validation phase									
	Protein microarray		Zhengzhou center			Nanchang center				Beijing center		
	HCC	NC	HCC	NC	CHB	HCC	NC	CHB	LC	HCC	CHB	LC
Sample size	54	27	261	261	66	91	138	21	91	61	17	61
Gender, n (%)												
Male	46 (85.2)	23 (85.2)	217 (83.1)	215 (82.4)	54 (82.0)	81 (89.0)	39 (28.3)	18 (85.7)	80 (87.9)	47 (77.0)	13 (76.5)	47 (77.0)
Female	8 (14.8)	4 (14.8)	44 (16.9)	45 (17.2)	11 (16.7)	10 (11.0)	99 (71.7)	3 (14.3)	11 (12.1)	13 (21.3)	3 (17.6)	13 (21.3)
Unknown	0	0	0	1 (0.4)	1 (1.3)	0	0	0	0	1 (1.7)	1 (5.9)	1 (1.7)
Age												
Range	40.1-78.3	20.1-70.7	28-87	18-87	31-82	20-80	23-78	27-71	23-76	32-85	41-79	31-81
Mean (SD)	56.8 (8.89)	53.3 (11.4)	56.2 (10.6)	56.0 (10.9)	55.9 (10.0)	54.7 (11.9)	45.8 (11.9)	53.2 (11.1)	53.0 (11.1)	58.1 (11.0)	57.7 (9.9)	50.0 (10.7)
Tumor size, n (%)												
≥ 3 cm	28 (51.9)		158 (60.5)			46 (50.5)				11 (18.0)		
< 3 cm	26 (48.1)		47 (18.0)			19 (20.9)				5 (8.2)		
Unknown	0		56 (21.5)			26 (28.6)				45 (73.8)		
Tumor number, n (%)												
Solitary	28 (51.9)		126 (48.3)			44 (48.4)				6 (9.8)		
Multiple	26 (48.1)		98 (37.5)			24 (26.4)				8 (13.1)		
Unknown	0		37 (14.2)			23 (25.3)				47 (77.1)		
TNM stage, n (%)												
Early stage	34 (63.0)		137 (52.5)			53 (58.2)				23 (37.7)		
Advanced	20 (37.0)		95 (36.4)			38 (41.8)				27 (44.3)		
Unknown	0		29 (11.1)			0				11 (18.0)		
Metastasis, n (%)												
Yes	7 (13.0)		28 (10.7)			11 (12.1)				6 (9.8)		
No	47 (87.0)		75 (28.7)			8 (8.8)				5 (8.2)		
Unknown			158 (60.5)			72 (79.1)				50 (82.0)		
AFP, n (%)												
Negative	27 (50.0)		88 (33.7)			27 (29.7)				25 (41.0)		
Positive	27 (50.0)		150 (57.5)			64 (70.3)				32 (52.5)		
Unknown	0		23 (8.8)			0				4 (6.5)		

HCC, hepatocellular carcinoma; NC, normal control; CHB, Chronic hepatitis B; LC, Liver Cirrhosis; SD, standard deviation; AFP, alpha-fetoprotein; AFP Positive, AFP ≥20 ng/mL; AFP Negative, AFP< 20 ng/ml.

### 3.2 High expression of anti-OLA1 autoantibody in HCC patients

**Figure 2:**
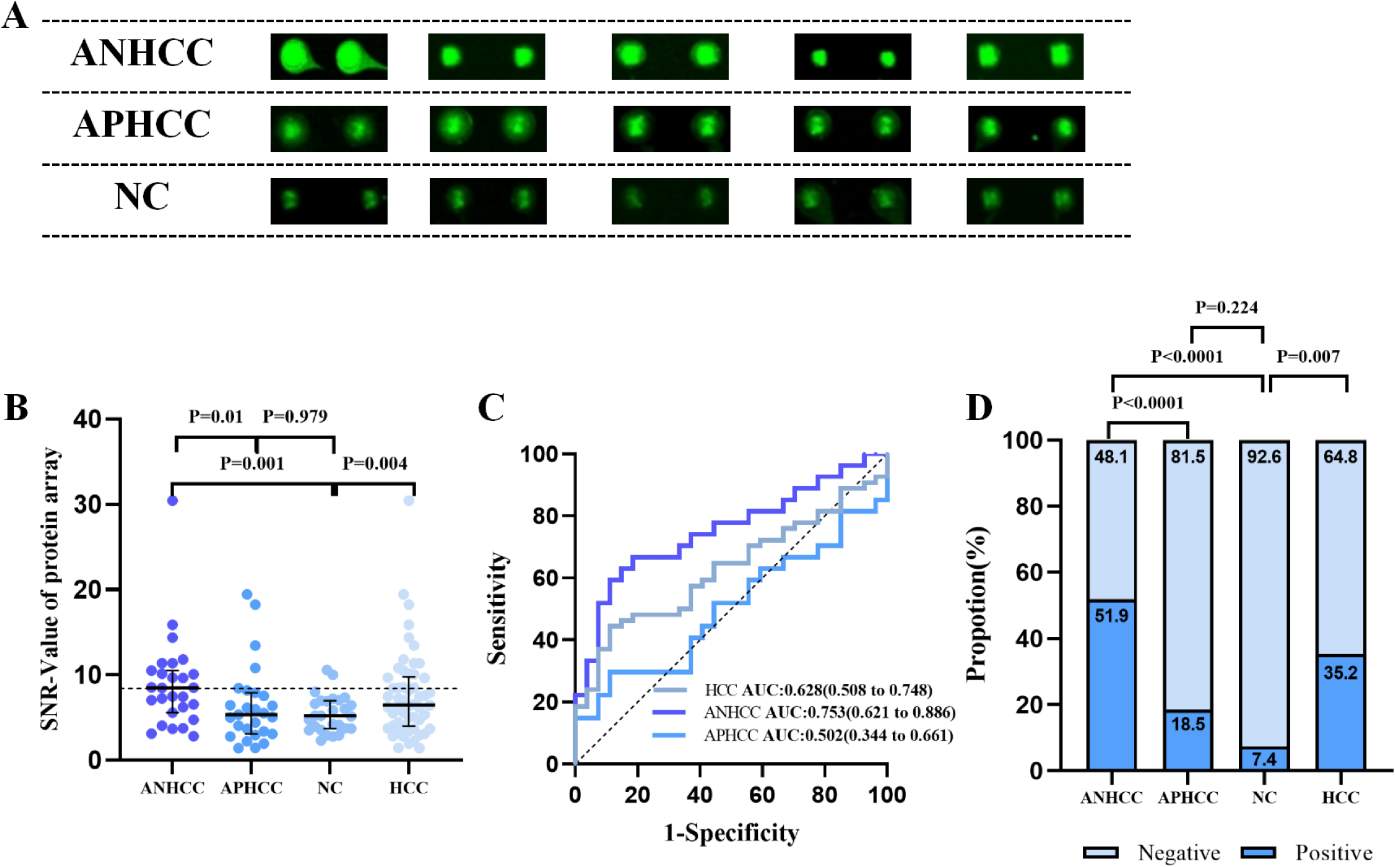
Anti-OLA1 autoantibody was discovered by using protein microarrays. **(A)** Microarray scan results of anti-OLA1 autoantibody in randomly selected ANHCC, APHCC, and NC samples. **(B)** scatter plot depicting SNR value of anti-OLA1 autoantibody across various groups. **(C)** The ROC of anti-OLA1 autoantibody in HCC, ANHCC, and APHCC. **(D)** Positive rates of anti-OLA1 autoantibody in different groups (the cutoff value was 8.43 determined by the 90% percentile of NC). ANHCC, AFP-negative hepatocellular carcinoma; APHCC, AFP-positive hepatocellular carcinoma; NC, Normal Control; SNR, the signal-to-noise ratio; AUC, area under receiver operator characteristic curve.

The protein microarray results revealed that the SNR of anti-OLA1 autoantibody was significantly higher in HCC patients than that in healthy controls. Moreover, within the HCC group, ANHCC patients exhibited a higher SNR of anti-OLA1 autoantibodies than APHCC patients (**Figures 2A, B**). Then, the receiver operating characteristic (ROC) curve analysis was performed. The results demonstrated that the area under the ROC curve (AUC) for ANHCC was 0.753, which is significantly higher than the AUC of 0.502 for APHCC (**Figure 2C**). Subsequently, the 90th percentile of the anti-OLA1 autoantibody SNR values in the healthy control population was adopted as the cutoff value, it was demonstrated that the sensitivity of anti-OLA1 autoantibody was 51.9% for ANHCC patients and 18.5% for APHCC patients (**Figure 2D**).

### 3.3 A multi-center validation of anti-OLA1 autoantibody as liquid biopsy biomarkers in HCC

**Figure 3:**
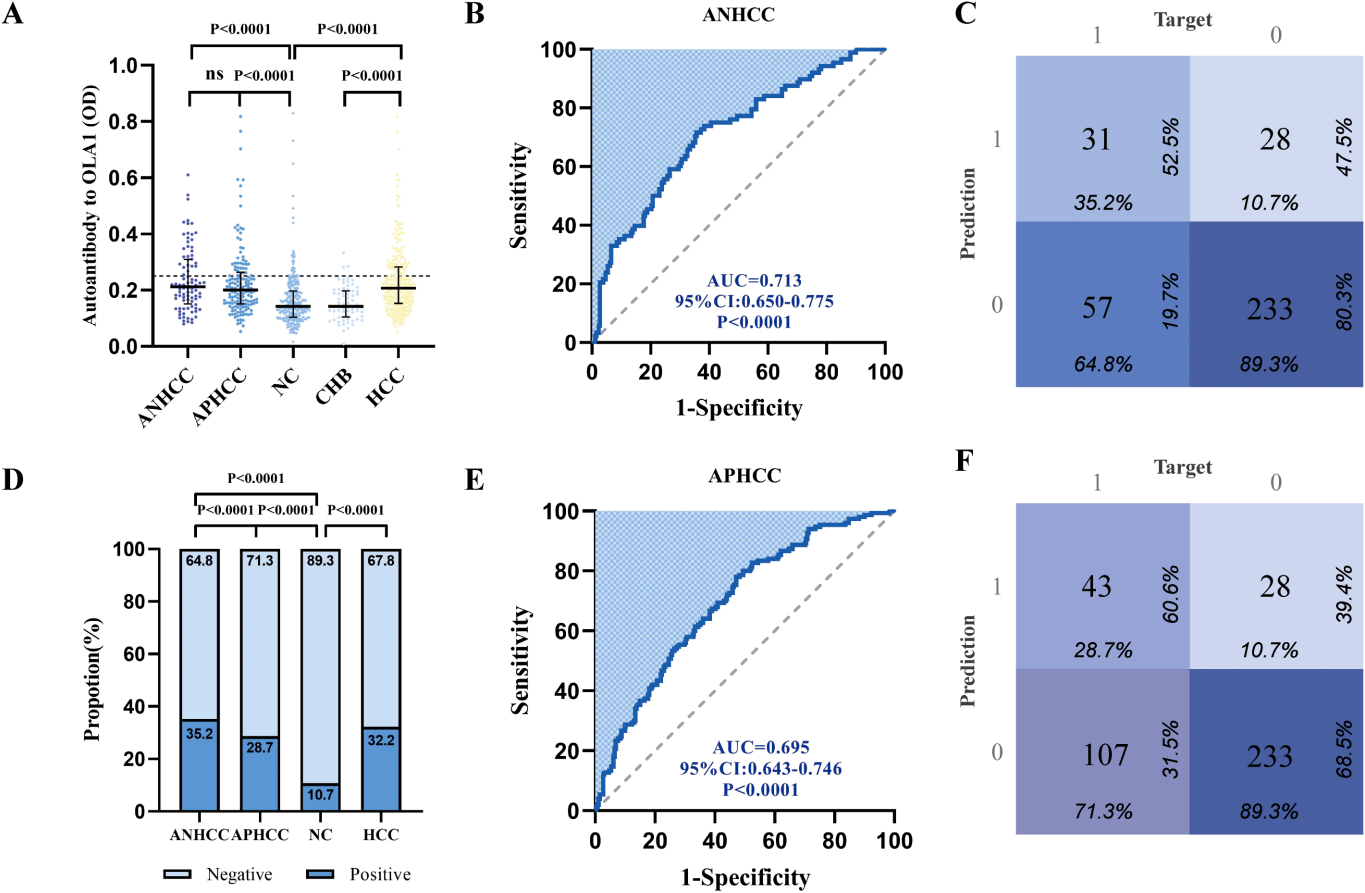
Multi-center validation of anti-OLA1 autoantibody. **(A)**the OD values of ELISA detection in ANHCC, APHCC, NC, and CHB groups. **(B, C)** ROC analysis and confusion matrix analysis for distinguishing ANHCC patients from NC in the Zhengzhou center. **(D)** Positive rates of anti-OLA1 autoantibody in different groups. **(E, F)** ROC analysis and confusion matrix analysis for distinguishing APHCC patients from NC in the Zhengzhou center. ANHCC, AFP-negative hepatocellular carcinoma; APHCC, AFP-positive hepatocellular carcinoma; NC, Normal Control; CHB, Chronic hepatitis B; LC, Liver Cirrhosis; ROC, the receiver operating characteristic.

To further investigate the diagnostic potential of anti-OLA1 autoantibody for HCC, we validated its diagnostic value across multiple center. Significantly elevated levels of anti-OLA1 autoantibody were observed in both ANHCC and APHCC patients compared to healthy controls or non-cancer controls across all three centers, with all *P*-values< 0.05. In the Zhengzhou center, serum optical density (OD) values measured by ELISA consistently demonstrated significant differences between HCC and non-HCC control groups (**Figure 3A**). The AUC value for distinguishing patients with ANHCC from healthy controls was 0.713, with a *P*-value<0.001 (**Figure 3B**). The confusion matrix of ANHCC was analyzed using the 90th percentile of the OD value of the healthy control group as the cut-off value. The results showed that its sensitivity was 35.2% and specificity was 89.3%, highlighting the excellent diagnostic performance of anti-OLA1 autoantibodies for ANHCC (**Figures 3C, D**). Similarly, the AUC value of APHCC also indicated its good diagnostic utility (AUC = 0.695) (**Figure 3E**), and a sensitivity of 28.7% for APHCC (**Figures 3D, F**). In the Nanchang center, AUC values for patients with ANHCC and APHCC compared to healthy controls were 0.672 and 0.656, respectively, resulting in sensitivities of 29.6% and 18.8%, alongside a specificity of 90.6% (**Supplementary Figure S1A**). In the center of Beijing, CHB and LC were categorized into the non-HCC group. The AUC values for ANHCC and APHCC in comparison to the non-HCC group were 0.607 and 0.624, respectively. The corresponding sensitivities were found to be 28.0% and 25.0% (**Supplementary Figure S1B**).

### 3.4 Universality of diagnosis of anti-OLA1 autoantibody in different clinical subgroups

An analysis of the diagnostic efficacy of anti-OLA1 autoantibody was conducted across different clinical subgroups, including metastasis status, tumor multiplicity, TNM stage, gender, and AFP status, due to the limited sample size in the Beijing center, data from the other two centers were used. The differences in anti-OLA1 autoantibody levels between healthy control and hepatitis control were compared, revealing no significant statistical differences between the two groups (**Supplementary Figure S2**). Consequently, the healthy and hepatitis populations were pooled as a non-cancer control for further analysis.

**Figure 4:**
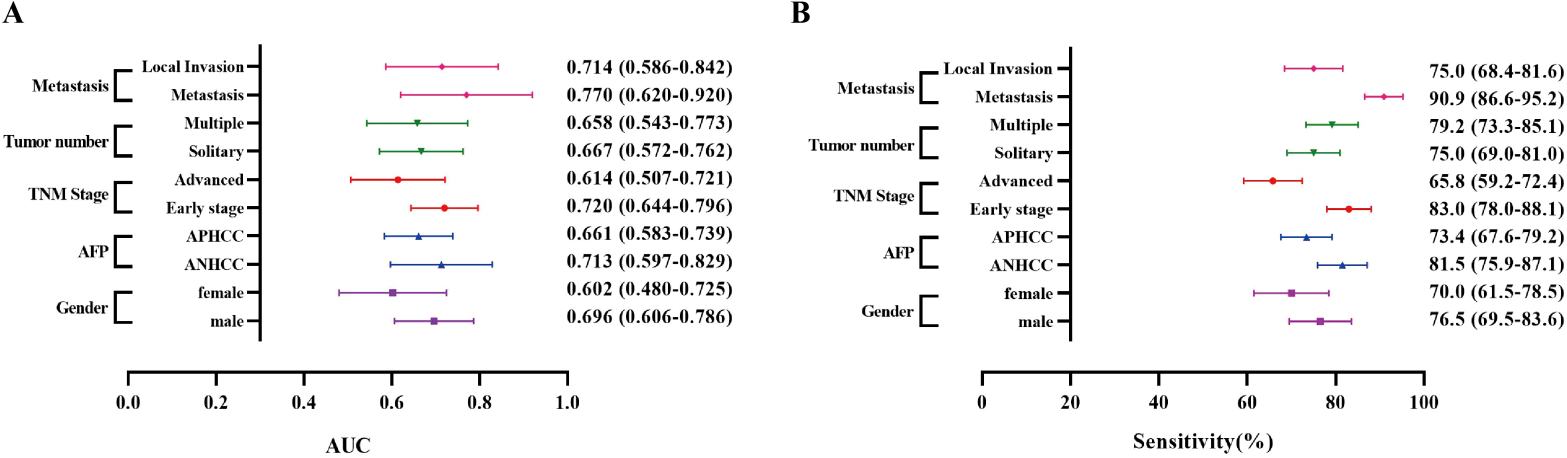
The AUCs and sensitivities of anti-OLA1 autoantibody in different subgroups from Zhengzhou Center. **(A)** The AUCs of the anti-OLA1 autoantibody in different subgroups. **(B)** The sensitivities of the anti-OLA1 autoantibody in different subgroups.

As a result, in the Zhengzhou center, the AUC ranged from 0.655 to 0.724, with sensitivities varying between 60.7% and 73.9% (**Figures 4A, B**, **Supplementary Table S2**). For the Nanchang center, the AUC range was found to be 0.602 - 0.770, and the sensitivity ranged from 65.8% to 90.9% (**Supplementary Figure S3**, **Supplementary Table S3**). The analysis of results from both centers revealed no significant differences in the diagnostic performance of anti-OLA1 autoantibody across various subgroups. This finding suggests the potential diagnostic universality of anti-OLA1 autoantibody.

### 3.5 The relationship between anti-OLA1 autoantibody and liver function parameters

During the development of HCC, the proliferation and invasion of tumor cells destroy normal liver cells, leading to impaired liver function. Consequently, the occurrence or progression of HCC is frequently associated with hepatic necrosis. So, we explored the correlation between the levels of anti-OLA1 autoantibody and some liver function parameters in sera. We analyzed a total of 416 serum samples from the Zhengzhou Center, 196 serum samples from the Nanchang Center, and 63 serum samples from the Beijing Center. All samples included complete liver function parameter information. Our findings revealed only a weak positive correlation between anti-OLA1 autoantibody levels and various liver function indicators, including Alanine Aminotransferase (ALT) (R = 0.17), Aspartate Aminotransferase (AST) with R of 0.25, Alkaline Phosphatase (ALP) (R = 0.14), and Gamma-Glutamyl Transpeptidase (GGT) (R = 0.17) (**Supplementary Figure S4**). Therefore, it was hypothesized that liver function parameters could complement anti-OLA1 autoantibody in the diagnosis of HCC.

Subsequently, we attempted to combine the aforementioned liver function-related indicators with anti-OLA1 autoantibody for HCC diagnosis using logistic regression. First, the samples from the Zhengzhou center were randomly split into two subsets at a ratio of 8:2, where 80% of the samples were used as the training set for model construction, and the remaining 20% were used as the test set for model validation. The formula of this diagnostic model is $P=\frac{1}{1+e^{-}(-9.378+4.577\times OLA1-1.374\times Log2ALT+1.771\times Log2AST+1.102\times Log2GGT)}$ . The event per variable (EPV) of this preliminary model was 43.25, which ensured the robustness of the model; meanwhile, no significant linear association was observed among the indicators as shown by collinearity analysis (**Supplementary Figure S5**).

**Figure 5:**
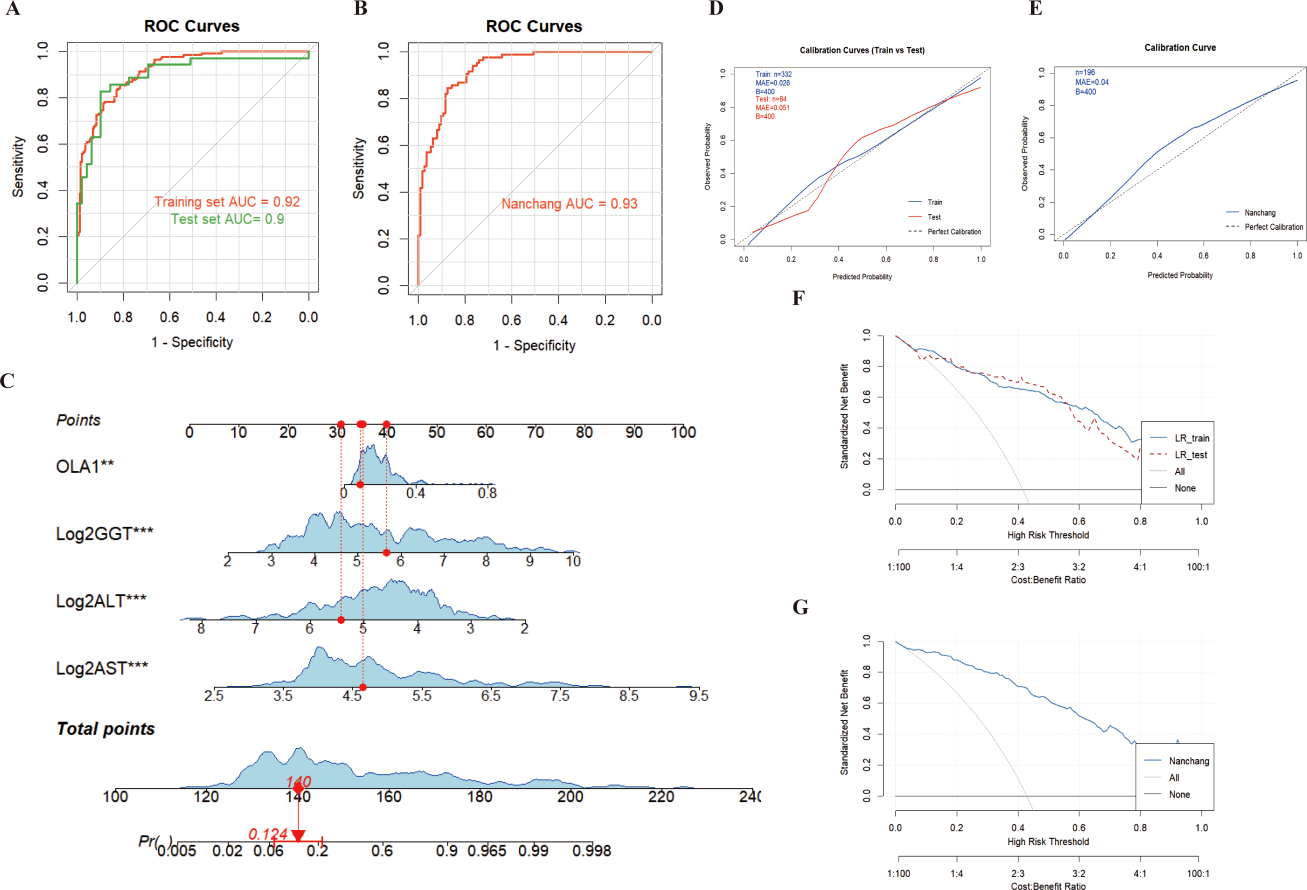
Diagnosis of HCC patients using anti-OLA1 autoantibody combined with partial liver function parameters. **(A**, **B)** ROC curve for joint diagnosis of Zhengzhou and Nanchang centers; **(C)** Nomogram of anti-OLA1 autoantibody combined with liver function parameters; **(D**, **E)** Calibration curves for Zhengzhou and Nanchang centers; **(F**, **G)** Decision curves for Zhengzhou and Nanchang centers.

The results from both the training set and the test set in the Zhengzhou center showed that ALT, AST and GGT were included in the model and could supplement the efficacy of anti-OLA1 autoantibody in the diagnosis of HCC, with an AUC of 0.92 (95% CI = 0.89–0.95) in the training set and 0.90 (95% CI = 0.82–0.97) in the test set (**Figure 5A**). Calibration curve evaluation and DCA curves further demonstrated the model’s stability and favorable clinical utility (**Figures 5D, F**). Compared with the diagnostic performance of anti-OLA1 autoantibody alone, the combined model yielded an NRI of 1.04 (training set) and 0.92 (test set), along with an IDI of 0.46 (training set) and 0.38 (test set) (**Supplementary Table S4**). Additionally, a nomogram was developed using the training set, which enables quick localization of scores corresponding to each variable of a patient, accumulates these scores to derive a total score, and thereby accurately predicts the probability of the patient developing HCC (**Figure 5C**).

Thereafter, validation conducted in the Nanchang Center also confirmed this finding, with AUC values of 0.93 (95% CI = 0.89–0.96) (**Figure 5B**). Additionally, the calibration curves and DCA curves of the model validated in the Nanchang Center exhibited stability (**Figures 5E, G**); for this validated model, the Net Reclassification Index (NRI) and Integrated Discrimination Index (IDI) were 0.88 and 0.46, respectively (**Supplementary Table S4**). At the Beijing Center, since no NC samples were included, we used HCC and CHB samples with complete liver function information to conduct combined modeling. The results also showed good diagnostic performance, with an AUC value of 0.88 (95% CI = 0.76–1.00). Furthermore, the combined validation model of the three centers (Zhengzhou, Nanchang, and Beijing) also enabled stable diagnosis of HCC, with an AUC of 0.92 (95% CI = 0.90–0.94) (**Supplementary Figure S6**).

### 3.6 The association between elevated anti-OLA1 autoantibody and OLA1 expression

**Figure 6:**
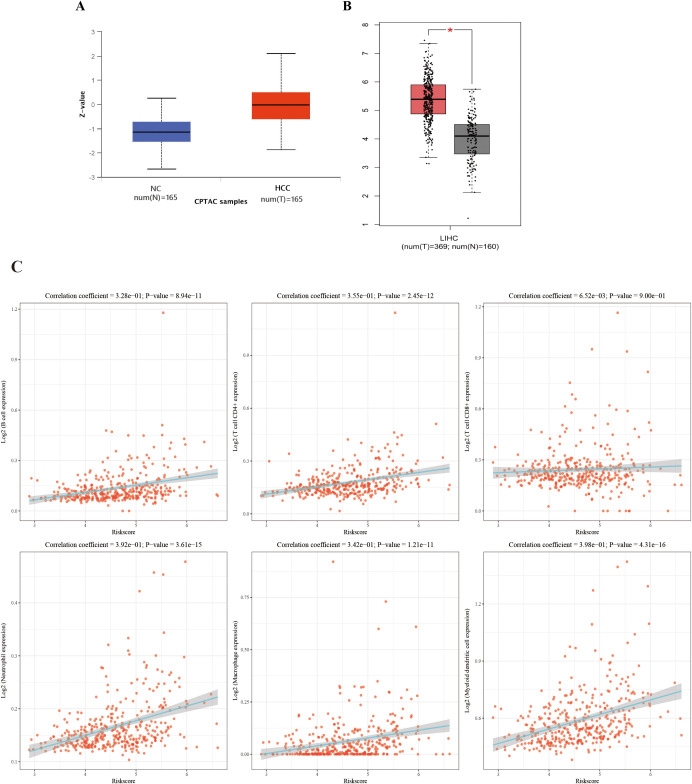
The correlation between OLA1 expression and immune cell infiltration in HCC. **(A)** Expression of OLA1 protein in HCC and NC in CPTAC database. **(B)** The mRNA expression of OLA1 in HCC and NC in TCGA database. **(C)** The correlation between OLA1 expression and immune cell infiltration in HCC. * denotes a statistically significant difference (*P* < 0.05) in mRNA expression levels between LIHC and NC samples when the |Log2 FC| cutoff is 1.0.

By querying the CPTAC and TCGA database, it was found that OLA1 is overexpressed in both protein and mRNA levels in HCC patients, which further supports the observation of elevated autoantibody levels against OLA1 (**Figures 6A, B**). Subsequently, to investigate the relationship between OLA1 and immune infiltration in HCC, the online webserver (http://timer.cistrome.org/) was used to analyze public liver cancer patient data from the TCGA database. The results revealed a significant positive correlation between OLA1 expression levels and the infiltration levels of multiple immune cells including B cells, CD4+T cells, neutrophils, macrophages, and dendritic cells (*P* < 0.05) (**Figure 6C**). This discovery suggests that OLA1 may play an important role in regulating immune responses within the HCC tumor microenvironment, potentially resulting in alterations of autoantibodies against this protein in the serum.

### 3.7 High expression of OLA1 in HCC is associated with poor prognosis

**Figure 7:**
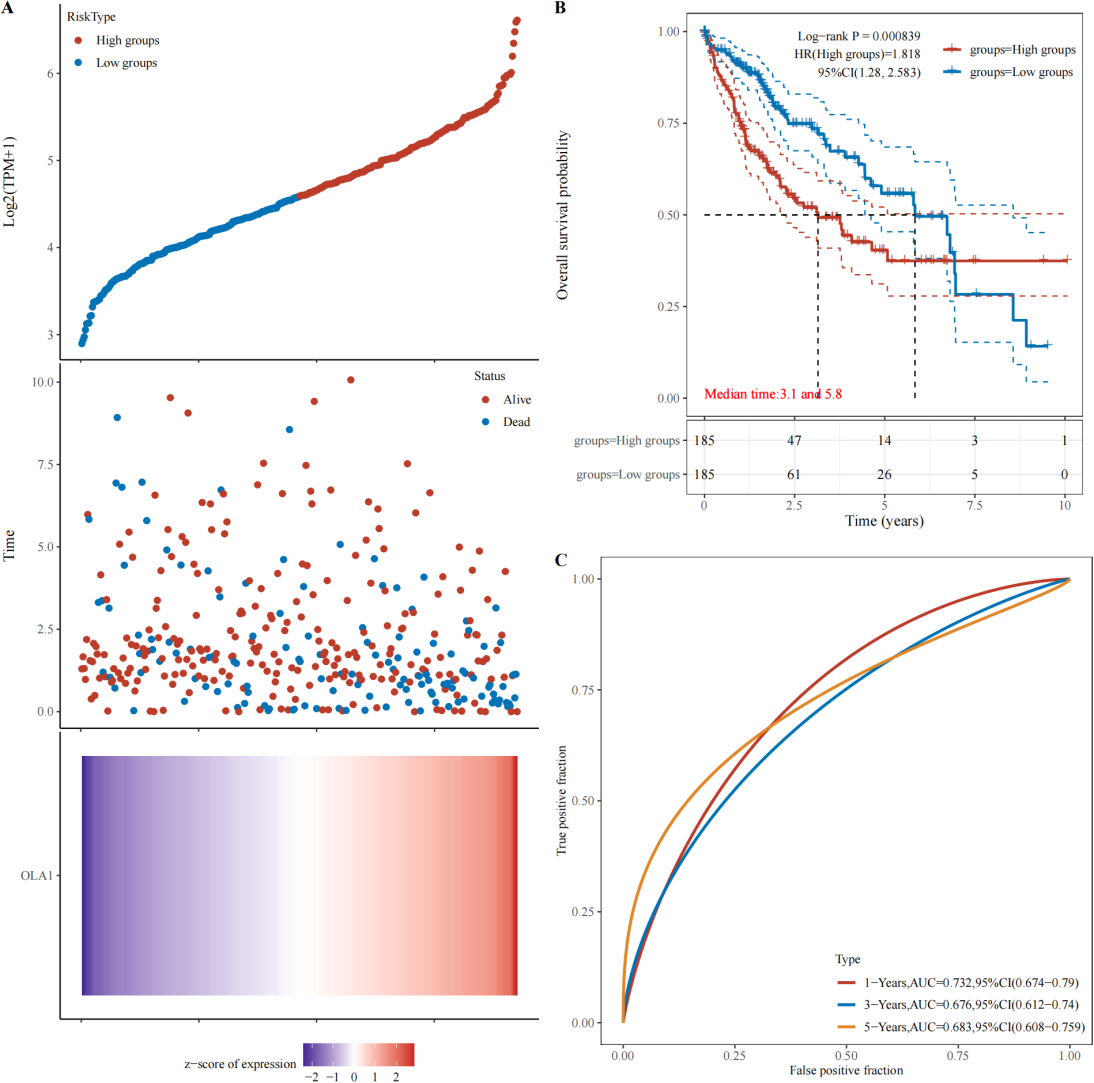
Relationship between OLA1 gene expression and prognosis. **(A)** The relationship between OLA1 gene expression and survival time and survival state in TCGA data. **(B)** K-M survival curve of OLA1 genes in TCGA data. **(C)** ROC curve and AUC value of genes at different times.

The webpage (https://www.aclbi.com/static/index.html#/) was used to analyze the relationship between OLA1 expression and HCC prognosis in the TCGA database. Based on OLA1 expression levels, patients were stratified into high-risk and low-risk groups, as time went by, the number of people in the high-expression group who were in “Alive” gradually decreased, and within the same period, the number of deaths in the high expression group is also higher compared to the low expression group (**Figure 7A**). Subsequently, Kaplan-Meier survival analysis was performed and a log-rank test was conducted to compare the survival distributions between the high-risk group and the low-risk group (**Figure 7B**). It revealed that higher OLA1 expression is significantly associated with poorer prognosis. Moreover, the efficacy of OLA1 expression levels in predicting the survival of HCC patients across different periods was evaluated. The AUC for one-year survival was 0.732 (**Figure 7C**), indicating that OLA1 can serve as a more accurate predictor of short-term survival risk in HCC patients.

## 4 Discussion

Many HCC patients are diagnosed in advanced stages, which markedly diminishes the efficacy of curative treatments (16). In the early stage of tumor development, autoantibodies exhibit higher sensitivity compared to their target antigens and can serve as potential biomarkers for the early diagnosis of HCC (10, 17–19). In this study, we identified that the level of anti-OLA1 autoantibody was significantly elevated in patients with HCC, particularly in those with ANHCC, compared to the NC group by protein microarray. Subsequently, a multicenter validation and evaluation was conducted and the results confirmed our expectations. The AUC for OLA1 autoantibody in distinguishing HCC from NC ranged from 0.607 to 0.713, with sensitivity ranging between 18.8% and 35.2%. These findings suggest that anti-OLA1 autoantibody has significant diagnostic value for patients with HCC.

In the context of screening tumor-associated autoantibodies, protein chip technology offers significant advantages. It facilitates high-throughput screening, thereby enabling the simultaneous detection of hundreds of autoantibodies (20). Furthermore, its exceptional sensitivity allows for the identification of autoantibodies even at very low concentrations (21, 22). In this study, we found that the anti - OLA1 autoantibody shows great potential in the diagnosis of HCC, especially in patients with ANHCC, as indicated by an AUC of 0.753. OLA1, or oncoprotein - induced transcript 1, is involved in various cellular processes such as cell cycle regulation and apoptosis. Abnormal expression of OLA1 has been associated with tumorigenesis (23, 24). Most previous research focused on the mRNA or protein levels of OLA1 (25). This is the first study to evaluate its corresponding autoantibody as a biomarker.

Subsequently, the results of the multi-center ELISA validation clearly demonstrated that the levels of anti-OLA1 autoantibody were significantly elevated in HCC patients at three clinical centers, and had higher sensitivity in ANHCC patients. The multicenter research approach we employed is a crucial element highlighted by Standards for the Reporting of Diagnostic Accuracy Studies (STARD), as it mitigates the potential biases inherent in single-center studies and enhances the generalizability of our findings (26, 27). By incorporating patients from three distinct clinical centers, we were able to account for variations in patient demographics, medical practices, and environmental factors that may influence the detection of anti-OLA1 autoantibody.

We conducted additional analyses on various clinical subgroups to validate the diagnostic performance of anti-OLA1 autoantibody. The findings indicated that anti-OLA1 autoantibodies exhibited robust diagnostic properties across subgroups with diverse characteristics, with AUC values ranging from 0.602 to 0.770. In various clinical subgroup analyses of tumor-associated autoantibodies, many findings are largely consistent with our research results on anti-OLA1 autoantibody. For instance, a comprehensive study assessed the diagnostic value of various tumor-associated autoantibodies in different subgroups of liver cancer. The researchers found that certain autoantibodies maintained similar diagnostic value across groups with varying clinical characteristics (11). While their sensitivity and specificity differed from those of anti-OLA1 autoantibody in our study, the functional patterns within each subgroup were quite similar. This further substantiates the potential of anti-OLA1 autoantibody as a stable and reliable biomarker for the diagnosis of HCC.

The liver, as an important organ with multiple physiological functions, plays a central role in metabolism and immune regulation. Abnormal liver function may potentially disrupt the balance of the immune system, leading to changes in the production and regulation of autoantibodies (28, 29). In our study, we found a subtle correlation (correlation coefficient R<0.3) between the levels of anti-OLA1 autoantibody and liver function parameters. Subsequently, we integrated the levels of anti-OLA1 autoantibody with liver function parameters using Logistic regression to explore their combined diagnostic efficacy for HCC. The results demonstrated a significant improvement in diagnostic efficacy, with AUC values of 0.92 and 0.93 for the Zhengzhou and Nanchang centers, respectively. Furthermore, these results also highlighted notable clinical net benefits. This discovery suggests that in the diagnosis of HCC, liver function parameters may exhibit a synergistic and complementary relationship with anti-OLA1 autoantibody. Numerous studies have revealed similar situations and underlying mechanisms, offering valuable insights for considering the potential interactions between autoantibodies and organ functions (30–32). Consequently, this enhances our understanding of the true rationale behind the diagnostic utility of anti-OLA1 autoantibodies in HCC.

OLA1 is significantly overexpressed in HCC patients according to the TCGA database and CPTAC, which may offer valuable insights into the potential mechanisms underlying the elevation of anti-OLA1 autoantibody. In addition, OLA1 expression is positively correlated with various immune cells in the HCC microenvironment, including B cells, CD4+T cells, neutrophils, macrophages, and dendritic cells, which strongly implies a complex regulatory network between autoantibodies and immune infiltration. It is noteworthy that B cells are crucial for antibody production (33). The positive correlation between OLA1 and B cells suggests that OLA1 overexpression in the liver cancer microenvironment may stimulate B cells to produce anti-OLA1 autoantibody. Consistent with previous studies (34, 35), the overexpression of protein may disrupt the tumor immune microenvironment and thereby altering the normal immune recognition mechanism. As a result, the immune system might misidentify OLA1 as a foreign antigen, promoting the production of anti-OLA1 autoantibody.

In terms of prognosis, high expression of OLA1 is associated with poorer survival outcomes in HCC patients. The AUC of one-year survival prediction is 0.732, indicating its potential value as a short-term prognostic indicator. This discovery further indicates that autoantibodies of OLA1 may serve as critical biomarkers for prognosis prediction, which will be the focus of our future research. Another limitation of this study is that the absence of AFP data in the control group prevented us from comparing the diagnostic performance of the anti-OLA1 autoantibody and our constructed model with that of AFP. Nevertheless, this study demonstrated that both the anti-OLA1 autoantibody and the model perform well in diagnosing ANHCC, supporting their utility as supplements to AFP for HCC detection.

In summary, our study demonstrates that the anti-OLA1 autoantibody is a potential biomarker for early diagnosis of HCC, especially in AFP-negative patients. The integration of anti-OLA1 autoantibody with liver function parameters shows an enhanced diagnostic efficacy for HCC. Additionally, the relationship between OLA1 and immune infiltration, as well as its prognosis, highlights its critical role in HCC pathogenesis and progression. Continued research in this area is expected to greatly enhance both diagnostic approaches and therapeutic strategies for HCC.

## Data Availability

The raw data supporting the conclusions of this article will be made available by the corresponding author. Additionally, the public data used in this article can be accessed via the https://ualcan.path.uab.edu/analysis and GEPIA databases (http://gepia.cancer-pku.cn/index.html).
