# Supplementary material for: Anti-OLA1 autoantibody is a potential early diagnostic marker for hepatocellular carcinoma
Source: Front Immunol. 2025 Oct 30;16:1647809. doi: 10.3389/fimmu.2025.1647809 (PMC12611910; doi:10.3389/fimmu.2025.1647809)
Supplement: Supplementary file 1 [file Table1.docx]

**Supplementary materials**

**Table S1** Information on the top 5 biomarkers in the protein microarray data.

|  | **SNR P50 (P25,P75)** | | **AUC** | **95% CI** |
| --- | --- | --- | --- | --- |
|  | **ANHCC (N=54)** | **NC (N=27)** |  |  |
| **OLA1** | 8.47 (5.59,10.51) | 5.21 (3.70,6.99) | 0.753 | 0.621-0.886 |
| **FOXL2** | 1.14 (1.03,1.28) | 1.03 (0.95,1.11) | 0.697 | 0.555-0.838 |
| **STAG2** | 1.20 (1.12,1.33) | 1.07 (1.04,1.24) | 0.627 | 0.494-0.759 |
| **CSF1R** | 1.53 (1.26,2.05) | 1.32 (1.17,1.57) | 0.626 | 0.500-0.751 |
| **PTEN** | 3.49 (1.59,6.93) | 1.60 (1.35,3.02) | 0.624 | 0.495-0.753 |

**Table S2** The diagnostic performance of anti-OLA1 autoantibody across various clinical groups in Zhengzhou center.

|  | **AUC (95%CI)** | ***P*** | **Se (%) (95%CI)** | **Sp (%) (95%CI)** | **PPV (%)** | **NPV (%)** | **Accuracy (%)** |
| --- | --- | --- | --- | --- | --- | --- | --- |
| **Gender, n (%)** |  |  |  |  |  |  |  |
| **Male (n = 586)** | 0.713 (0.668-0.759) | <0.0001 | 71.0 (66.9-75.0) | 59.5 (55.1-63.8) | 58.3 | 71.7 | 64.6 |
| **Female (n = 100)** | 0.672 (0.566-0.778) | 0.003 | 65.9 (56.6-75.2) | 62.5 (53.0-72.0) | 58.0 | 70.0 | 64.0 |
| **AFP, n (%)** |  |  |  |  |  |  |  |
| **Negative (n = 88)** | 0.715 (0.654-0.776) | <0.0001 | 73.9 (69.6-78.1) | 60.2 (55.5-65.0) | 33.3 | 89.5 | 63.1 |
| **Positive (n = 150)** | 0.698 (0.648-0.747) | <0.0001 | 66.7 (62.4-70.9) | 60.2 (55.9-64.6) | 43.5 | 79.8 | 62.3 |
| **TNM stage, n (%)** |  |  |  |  |  |  |  |
| **Early stage(n = 137)** | 0.711 (0.661-0.762) | <0.0001 | 69.3 (65.1-73.5) | 60.2 (55.8-64.7) | 42.2 | 82.4 | 62.9 |
| **Advanced (n = 95)** | 0.724 (0.667-0.782) | <0.0001 | 72.6 (68.4-76.9) | 60.2 (55.6-64.9) | 34.7 | 88.3 | 63.0 |
| **Tumor number, n (%)** |  |  |  |  |  |  |  |
| **Solitary(n = 126)** | 0.721 (0.670-0.773) | <0.0001 | 71.4 (67.3-75.6) | 60.2 (55.7-64.8) | 40.9 | 84.5 | 63.4 |
| **Multiple(n = 98)** | 0.699 (0.640-0.758) | <0.0001 | 68.4 (63.9-72.8) | 60.2 (55.6-64.9) | 34.0 | 86.4 | 62.1 |
| **Metastasis, n (%)** |  |  |  |  |  |  |  |
| **Yes (n = 28)** | 0.655 (0.557-0.752) | 0.007 | 60.7 (55.6-65.8) | 60.2 (55.2-65.3) | 11.6 | 94.7 | 60.3 |
| **No (n = 75)** | 0.720 (0.657-0.783) | <0.0001 | 73.7 (69.0-77.7) | 60.2 (55.5-65.0) | 29.7 | 90.8 | 62.7 |

Note:

Cut off: 0.163 was determined by the maximum Youden index of the ROC curve for the Zhengzhou sample. Se, sensitivity; Sp, specificity; PPV, positive predictive value; NPV, negative predictive value.

**Table S3**.The diagnostic performance of the validation cohort in Nanchang center.

|  | **AUC (95%CI)** | ***P*** | **Se (%) (95%CI)** | **Sp (%) (95%CI)** | **PPV (%)** | **NPV (%)** | **Accuracy (%)** |
| --- | --- | --- | --- | --- | --- | --- | --- |
| **Gender, n (%)** |  |  |  |  |  |  |  |
| **Male (n = 586)** | 0.696 (0.606-0.786) | <0.0001 | 76.5 (69.5-83.6) | 56.1 (47.9-64.4) | 71.3 | 62.7 | 68.1 |
| **Female (n = 100)** | 0.602 (0.480-0.725) | 0.286 | 70.0 (61.5-78.5) | 56.9 (47.7-66.0) | 13.7 | 95.1 | 58.0 |
| **AFP, n (%)** |  |  |  |  |  |  |  |
| **Negative (n = 88)** | 0.713 (0.597-0.829) | <0.0001 | 81.5 (75.9-87.1) | 56.6 (49.5-63.7) | 24.2 | 54.7 | 60.2 |
| **Positive (n = 150)** | 0.661 (0.583-0.739) | <0.0001 | 73.4 (67.6-79.2) | 56.6 (50.1-63.1) | 40.5 | 84.1 | 61.4 |
| **TNM stage, n (%)** |  |  |  |  |  |  |  |
| **Early stage(n = 137)** | 0.720 (0.644-0.796) | <0.0001 | 83.0 (78.0-88.1) | 56.6 (49.9-63.3) | 38.9 | 90.9 | 63.2 |
| **Advanced (n = 95)** | 0.614 (0.507-0.721) | 0.029 | 65.8 (59.2-72.4) | 56.6 (49.7-63.5) | 26.6 | 87.4 | 58.4 |
| **Tumor number, n (%)** |  |  |  |  |  |  |  |
| **Solitary (n = 126)** | 0.667 (0.572-0.762) | 0.001 | 75.0 (69.0-81.0) | 56.6 (49.8-63.4) | 32.4 | 89.1 | 60.6 |
| **Multiple (n = 98)** | 0.658 (0.543-0.773) | 0.013 | 79.2 (73.3-85.1) | 56.6 (49.4-63.8) | 21.6 | 94.7 | 59.6 |
| **Metastasis, n (%)** |  |  |  |  |  |  |  |
| **Yes (n = 28)** | 0.770 (0.620-0.920) | 0.003 | 90.9 (86.6-95.2) | 56.6 (49.2-64.1) | 12.7 | 98.9 | 58.8 |
| **No (n = 75)** | 0.714 (0.586-0.842) | 0.042 | 75.0 (68.4-81.6) | 56.6 (49.1-64.1) | 8.0 | 97.8 | 57.5 |

Note:

Cut off: 0.173 was determined by the maximum Jordan index of the ROC curve for the Nanchang sample. Se, sensitivity; Sp, specificity; PPV, positive predictive value; NPV, negative predictive vaule.

**Table S4.** Analysis of NRI and IDI after combined modeling of anti-OLA1 autoantibody and liver function parameters.

|  | NRI | *P* | IDI | *P* |
| --- | --- | --- | --- | --- |
| Zhengzhou Center |  |  |  |  |
| Training set | 1.04 | <0.0001 | 0.46 | <0.0001 |
| Test set | 0.92 | <0.0001 | 0.38 | <0.0001 |
| Nanchang Center | 0.88 | <0.0001 | 0.46 | <0.0001 |
| Beijing Center | 0.78 | 0.004 | 0.39 | <0.0001 |


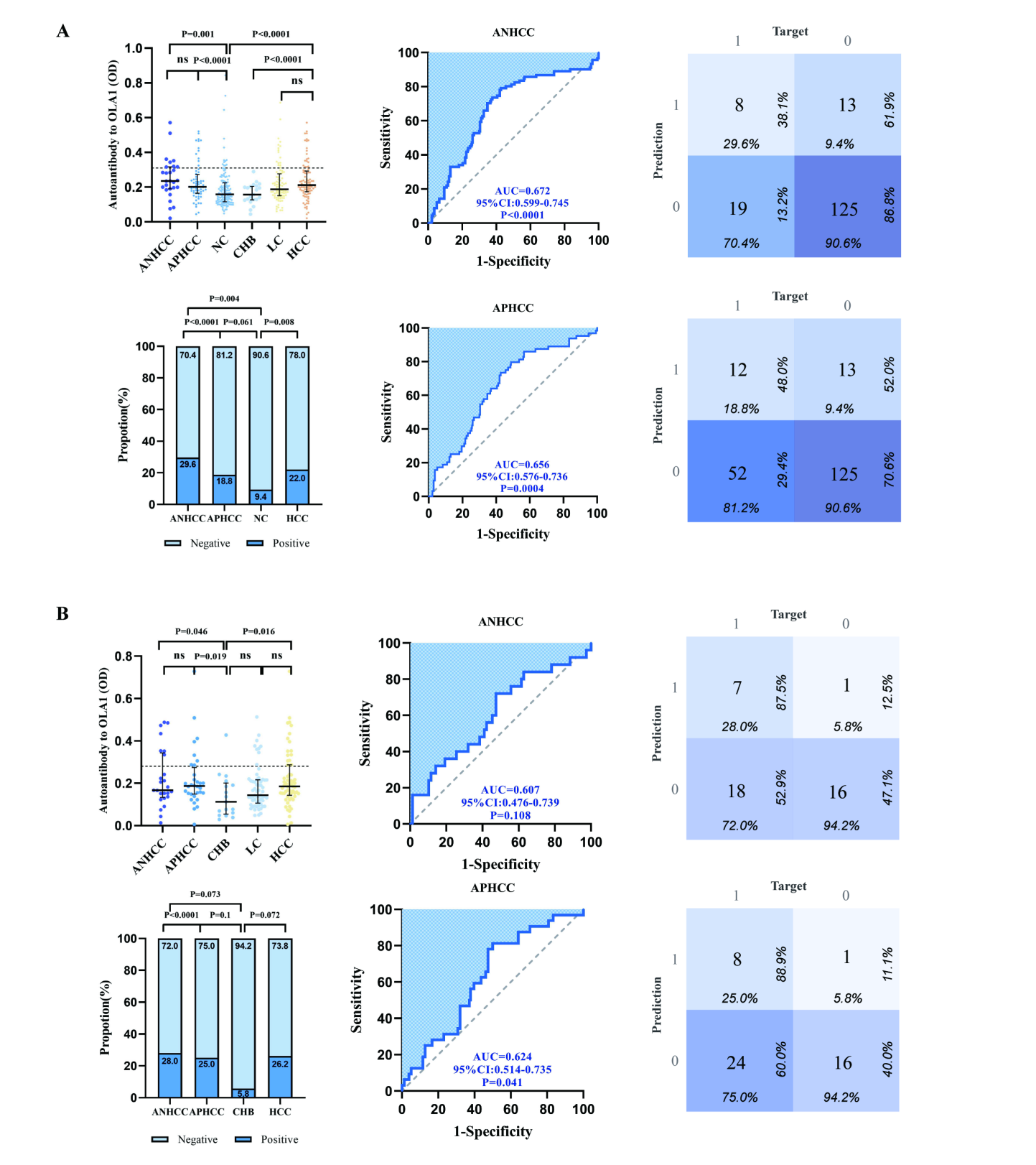


**Fig S1** ROC, confusion matrix, and sensitivity specificity analysis were performed on HCC and NC samples from Nanchang Center and Beijing Center. **A** OD values detected by ELISA at Nanchang Center, ROC analysis and confusion matrix analysis of ANHCC and APHCC patients and NC controls. **B** OD values detected by ELISA at Beijing Center, ROC analysis and confusion matrix analysis of ANHCC and APHCC patients and NC controls. ANHCC, AFP-negative hepatocellular carcinoma; APHCC, AFP-positive hepatocellular carcinoma；NC, Normal Control; CHB, Chronic hepatitis B; LC, Liver Cirrhosis; ROC, the receiver operating characteristic.


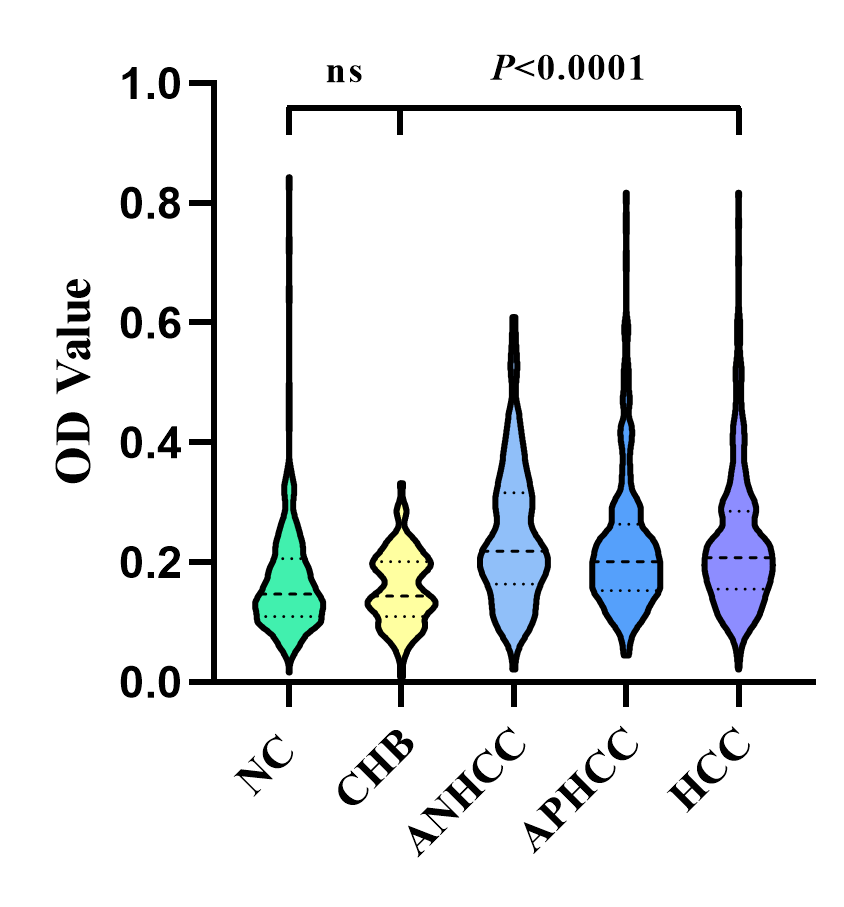


**Fig S2**. Violin charts of HCC, NC, and CHB in Zhengzhou and Nanchang centers.


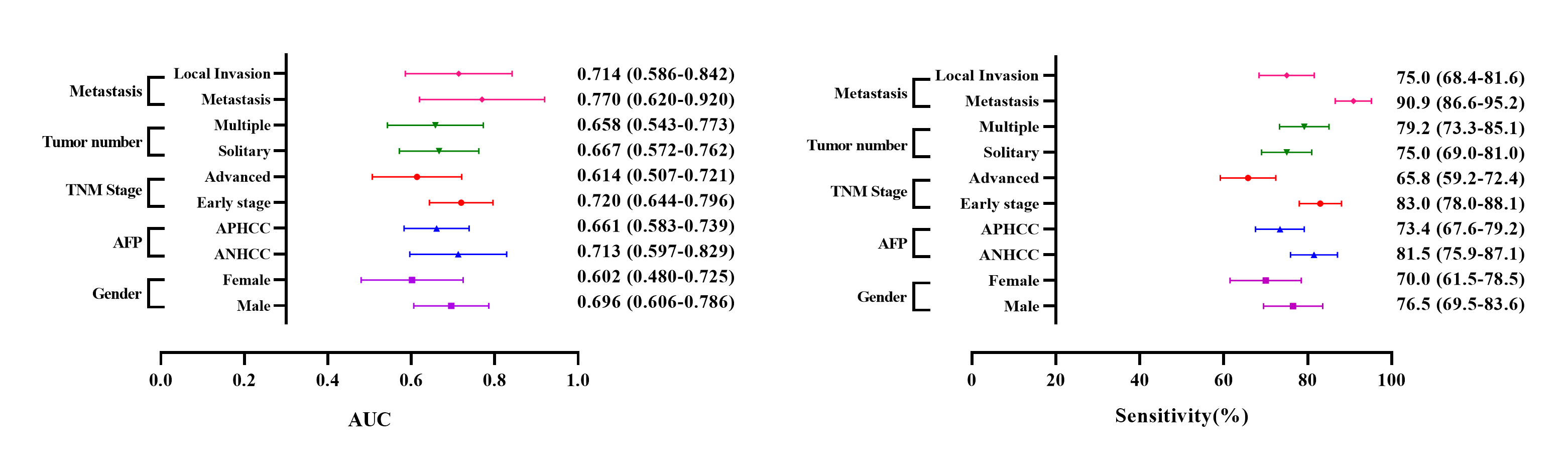


**Fig S3**. Clinical AUC and sensitivity of anti-OLA1 autoantibody in different subgroups of Nanchang Center. **A** The AUC of the anti-OLA1 autoantibody in different subgroups. **B** The sensitivity of the anti-OLA1 autoantibody in different subgroups.


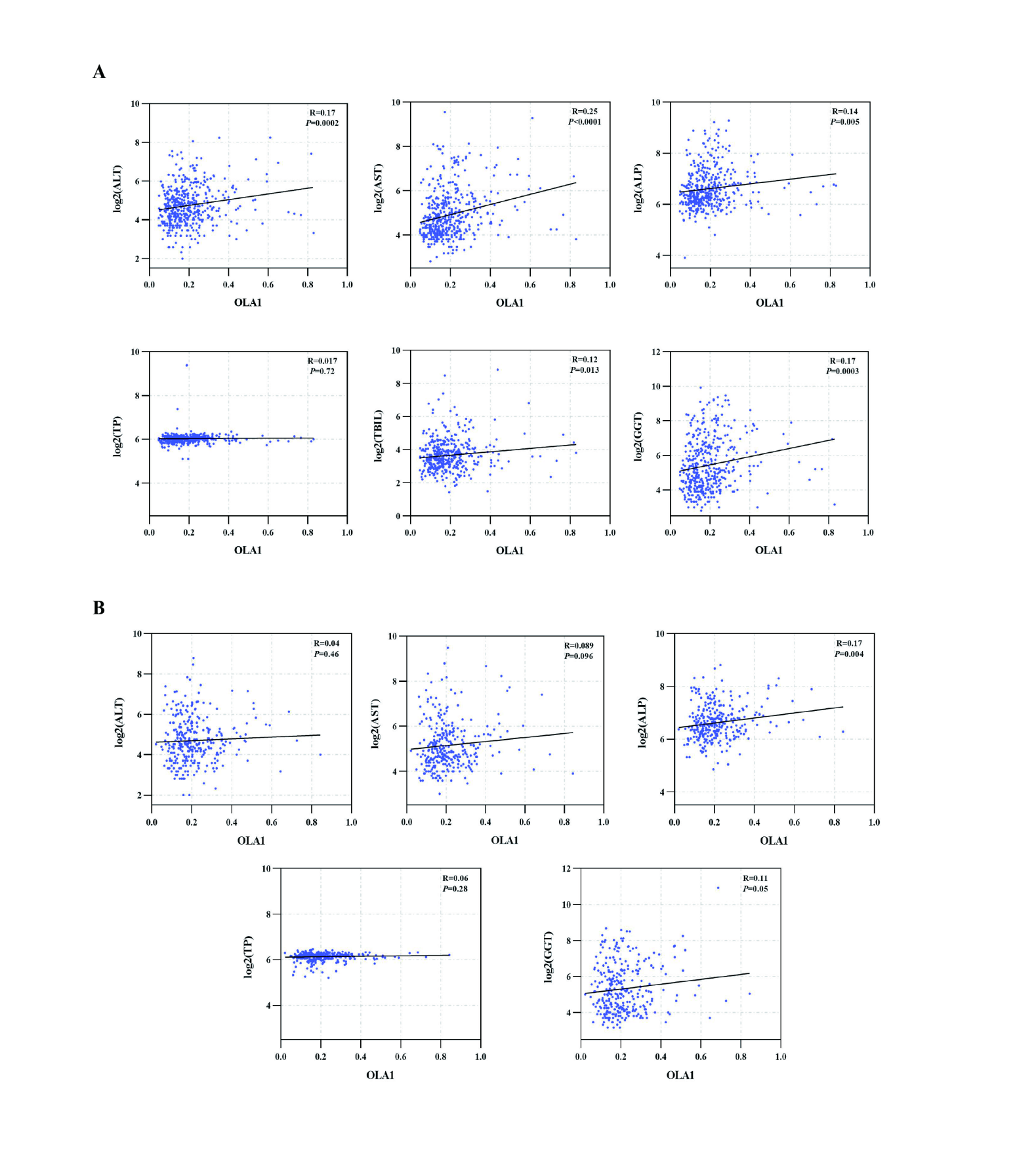


**Fig S4**. The correlation between the expression level of anti-OLA1 autoantibody and different liver function parameters in Zhengzhou and Nanchang Centers.


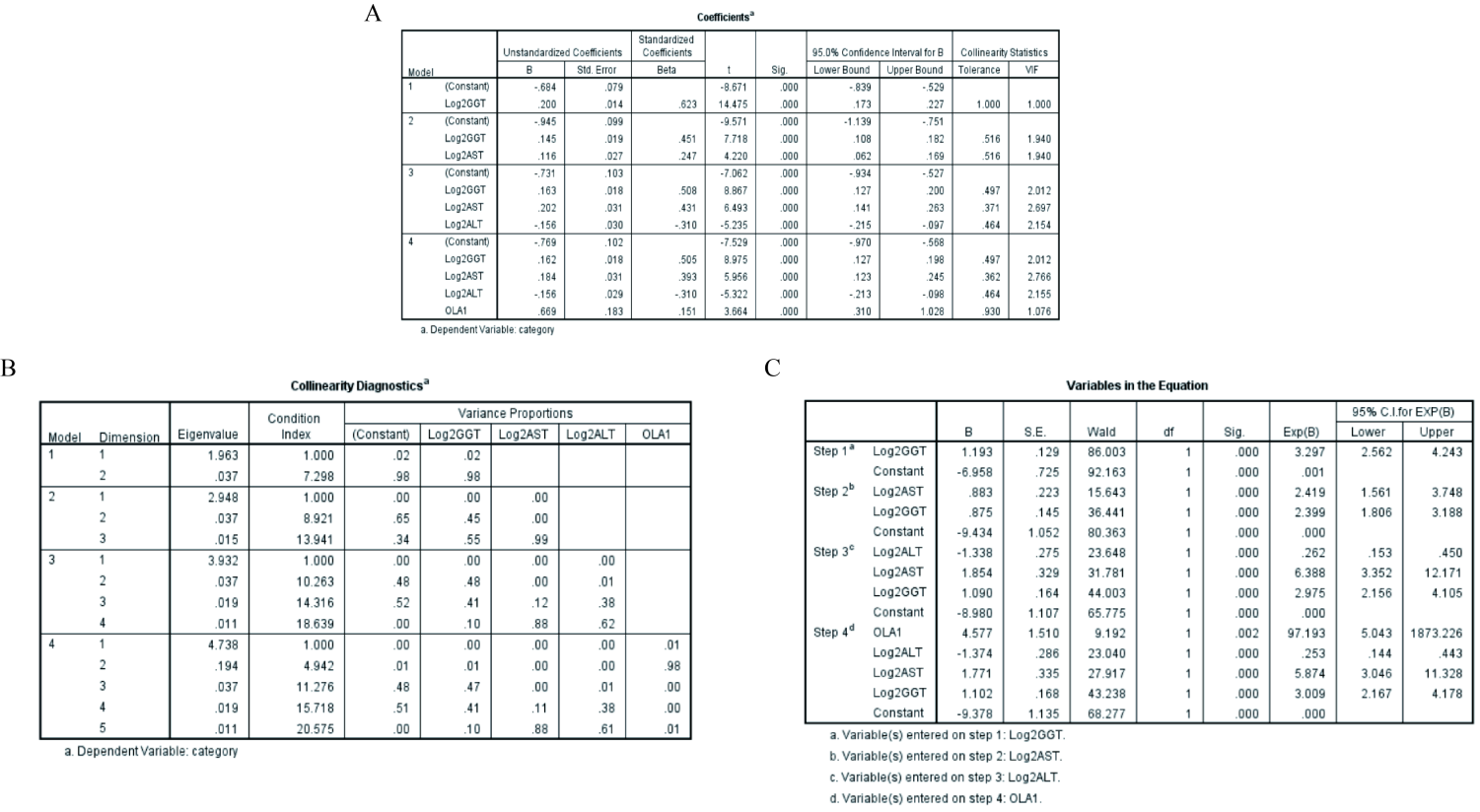


**Fig S5**. The indicator contribution and indicator collinearity status of the Logistic Regression Model. **A** Comprises “Coefficients” and “Collinearity Diagnostics”. The “Coefficients” section presents regression coefficients and collinearity statistics (Tolerance, VIF), indicating weak collinearity among independent variables; the “Collinearity Diagnostics” section further verifies the absence of obvious collinearity via eigenvalues, condition indices, and variance proportions, ensuring the stability of coefficient estimation in the model. **B** “Collinearity Diagnostics” (detailed table) supplements variance proportion information to further confirm no severe multicollinearity. **C** “Variables in the Equation” illustrates the significant predictive effects, odds ratios (Exp(B)), and stepwise entry process.


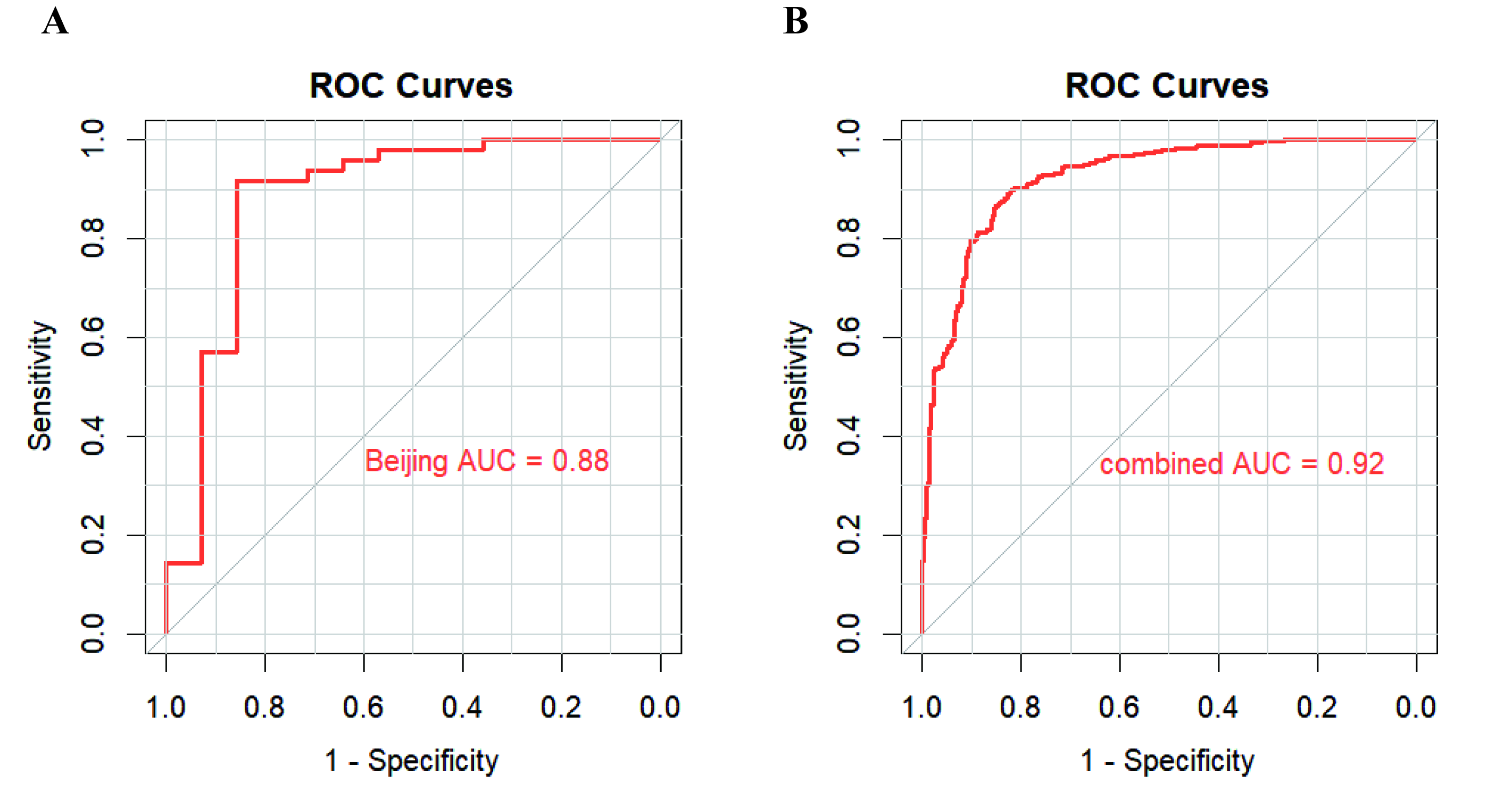


**Fig S6**. Correlation data analysis of anti-OLA1 autoantibody combined with liver function parameters in constructing a diagnostic model. **A** ROC curve for the diagnosis of HCC by anti-OLA1 autoantibody combined with liver function parameters in the Beijing center; **B** ROC curve for the diagnosis of HCC by the multicenter combined model.
